# Analysis of Methamphetamine‐Related Deaths: A Retrospective Cross‐Sectional Study

**DOI:** 10.1111/dar.70241

**Published:** 2026-08-17

**Authors:** Arif Garbioğlu, Hacı Altay

**Affiliations:** ^1^ Department of Forensic Medicine, Faculty of Medicine Zonguldak Bulent Ecevit University Zonguldak Turkey; ^2^ Zonguldak Forensic Medicine Branch Office Council of Forensic Medicine Zonguldak Turkey

**Keywords:** forensic autopsy, forensic toxicology, methamphetamine‐related deaths, postmortem analysis, toxicity

## Abstract

**Introduction:**

Methamphetamine use and related mortality have increased worldwide, yet forensic data from Turkey remain limited.

**Methods:**

This retrospective cross‐sectional autopsy study investigated the demographic, toxicological and medicolegal characteristics of methamphetamine‐related deaths in Zonguldak, Turkey, between 2015 and 2025. Among the 1863 forensic autopsies, 30 cases with analytically confirmed methamphetamine positivity were included.

**Results:**

The mean age was 33.4 ± 8.7 years and 90.0% of the cases were male. The highest annual proportion of forensic autopsies with methamphetamine detected was observed in 2023. Accidental death was the most common manner of death and methamphetamine toxicity was the leading cause of death. All natural deaths were attributed to cardiovascular disease. Polydrug use was present in 56.7% of the cases.

**Discussion and Conclusions:**

Our findings indicate that methamphetamine‐related deaths in Turkey share demographic and forensic characteristics with international reports and underscore the growing medicolegal and public health burden associated with stimulant use.

## Summary

- Methamphetamine detection in forensic autopsies in Zonguldak was uncommon but observed across the study period, with annual counts fluctuating.
- Most methamphetamine‐related deaths involved young adult men and were associated with social vulnerability and psychiatric comorbidities.
- Toxicological methamphetamine concentrations should be interpreted in conjunction with clinical circumstances, autopsies and contextual findings.

## 1 Introduction

The global rise in substance use continues to represent a major public health challenge. According to the World Drug Report 2025, approximately 316 million individuals worldwide used drugs in 2023, marking a sustained increase over the past decade that has outpaced population growth and signals an expanding global burden of drug use [1].

Methamphetamine (MA) and amphetamine‐type stimulants remain the dominant synthetic drugs globally. Recorded seizure levels in 2023 indicate increased availability. An estimated 31 million people worldwide use these substances, and MA accounted for the largest share of both use and trafficking [1].

MA use has been associated with violent behaviour and an increased risk of non‐natural deaths, including traffic‐related accidents, suicides and homicides [2, 3]. Empirical evidence further indicates that mortality among people who use MA is substantially elevated and is estimated to be 3 to 6 times higherthan that among people who do not use MA [4]. In Turkey, MA remains the most commonly used amphetamine‐type stimulant, with a continued sharp increase in seizures, increasing by 54.4% in 2024 compared with 2023. This trend is paralleled by its increasing impact on mortality, as MA has been identified as one of the leading substances involved in direct drug‐related deaths, second only to synthetic cannabinoids [5].

Turkey is situated along one of the major drug routes of trafficking. Its strategic position—combined with extensive land, sea and air connections facilitating legitimate international trade—has contributed to evolving patterns of illicit drug trafficking and smuggling [1].

Evidence on MA‐related deaths in Turkey remains scarce. Although previous studies have described the toxicological, sociodemographic and epidemiological characteristics of drug‐related deaths [6, 7], and MDMA‐related deaths in Turkey [8], data specifically focusing on MA‐related deaths are limited. Therefore, this study aims to investigate the patterns and characteristics of MA‐related deaths in an adult forensic population in Zonguldak, Turkey, between 2015 and 2025.

## 2 Methods

### 2.1 Study Design

This single‐centre study was conducted in Zonguldak, a city with a population of 585,203 [9], which is located in the westernmost part of the Western Black Sea region of Turkey. The city plays a significant role in maritime trade between Turkey and other Black Sea countries because of its strategic port [10].

This retrospective study analysed 1863 autopsies performed at the Zonguldak Forensic Medicine Branch Office between 2015 and 2025 under orders from public prosecutor offices. Among these cases, 30 met the inclusion criteria.

This study was conducted in compliance with the ethical principles according to the Declaration of Helsinki and was approved by the Non‐Interventional Clinic Research Ethics Committee of the Zonguldak Bulent Ecevit University (No. 2025/18, Date. 15 October 2025). All data were anonymised and handled with strict confidentiality.

### 2.2 Definitions

In Turkey, all deaths suspected to be related to drug use are classified as medicolegal cases and are therefore subject to postmortem examination. In such cases, comprehensive toxicological and anatomical evaluations are performed, including full autopsies as well as gross and histopathological examinations of organs. Comprehensive toxicological and chemical analyses are performed on blood, urine, vitreous humour, gastric contents, bile and organ samples, screening for ethanol, methanol, organophosphates, organochlorines, carbamates, carboxyhemoglobin, illicit drugs and a broad range of medications.

During autopsy, biological specimens were collected and submitted to the Council of Forensic Medicine's forensic toxicology laboratories, which have been accredited under the international standard ISO/IEC 17025 since 2009 [11]. The samples were analysed via systematic toxicological methods routinely applied in drug screening protocols in these laboratories. All findings were verified via multiple analytical techniques to obtain quantitative results for each detected compound. Initial screening was performed via gas chromatography–mass spectrometry and liquid chromatography–quadrupole time‐of‐flight mass spectrometry for a broad range of drugs. Confirmatory analyses were conducted via liquid chromatography–tandem mass spectrometry. The quantitative analysis of alcohols was performed via headspace gas chromatography with flame ionisation detection. Blood carboxyhemoglobin levels were measured via a CO‐Oximeter.

Study data were derived from premortem clinical records, medical and circumstantial history obtained from relatives and documented in police and public prosecutor's investigation files, witness statements, autopsy reports, toxicological analyses and histopathological examination findings.

### 2.3 Measures

All cases between 2015 and 2025 with analytically confirmed MA detection in postmortem toxicological samples were included, whereas cases with positive findings unrelated to MA were excluded. MA concentrations were measured in peripheral blood samples. Paediatric and foetal cases were also excluded to ensure a homogeneous and comparable study population, given the substantial differences in exposure patterns and pathophysiology compared with those of adults [12, 13].

The collected variables included sociodemographic characteristics—such as age, sex, weight, height, marital status, living conditions, education and employment status—when available. In addition, histories of psychiatric illness, illicit drug use and alcohol consumption were recorded as reported or not reported. The incident‐related variables included year, time of the incident (00:00–05:59, 06:00–11:59, 12:00–17:59 and 18:00–23:59), incident location, manner and cause of death, location of death and medical intervention status.

Cases were categorised according to the certified cause and manner of death following completion of the medico‐legal investigation. The classification was based on the combined evaluation of scene investigation findings, police and prosecutor records, premortem clinical information, complete autopsy findings, histopathological examination and toxicological analyses. Cases were classified as MA toxicity deaths when MA was determined to be the primary cause of death after comprehensive forensic evaluation. Cases in which MA was detected but another disease or injury was determined to be the primary cause of death were classified according to that primary cause of death. The recorded causes of death included MA toxicity, asphyxia by hanging, injuries (traffic injury, firearm injury, sharp force injury, electrical injury, fall from height, fire‐related injury) and cardiovascular disease. The manner of death was classified as natural, accidental, suicidal or homicidal.

The autopsy‐related variables included autopsy findings, toxicological results and histopathological results. All the data were anonymised prior to analysis.

### 2.4 Statistical Analysis

The data were analysed via SPSS 29.0 (IBM Corp., Armonk, NY, USA). Continuous data were stated as the means ± standard deviations and medians with quartiles (Q1–Q3), whereas categorical data were presented as frequencies (*n*) and percentages (%). Given the skewed distribution and presence of outliers in MA concentrations, median values were reported for these data. Given the retrospective descriptive design, the limited sample size and the absence of a control group, no inferential comparative analyses were performed. The findings were summarised using descriptive statistics.

## 3 Results

**FIGURE 1:**
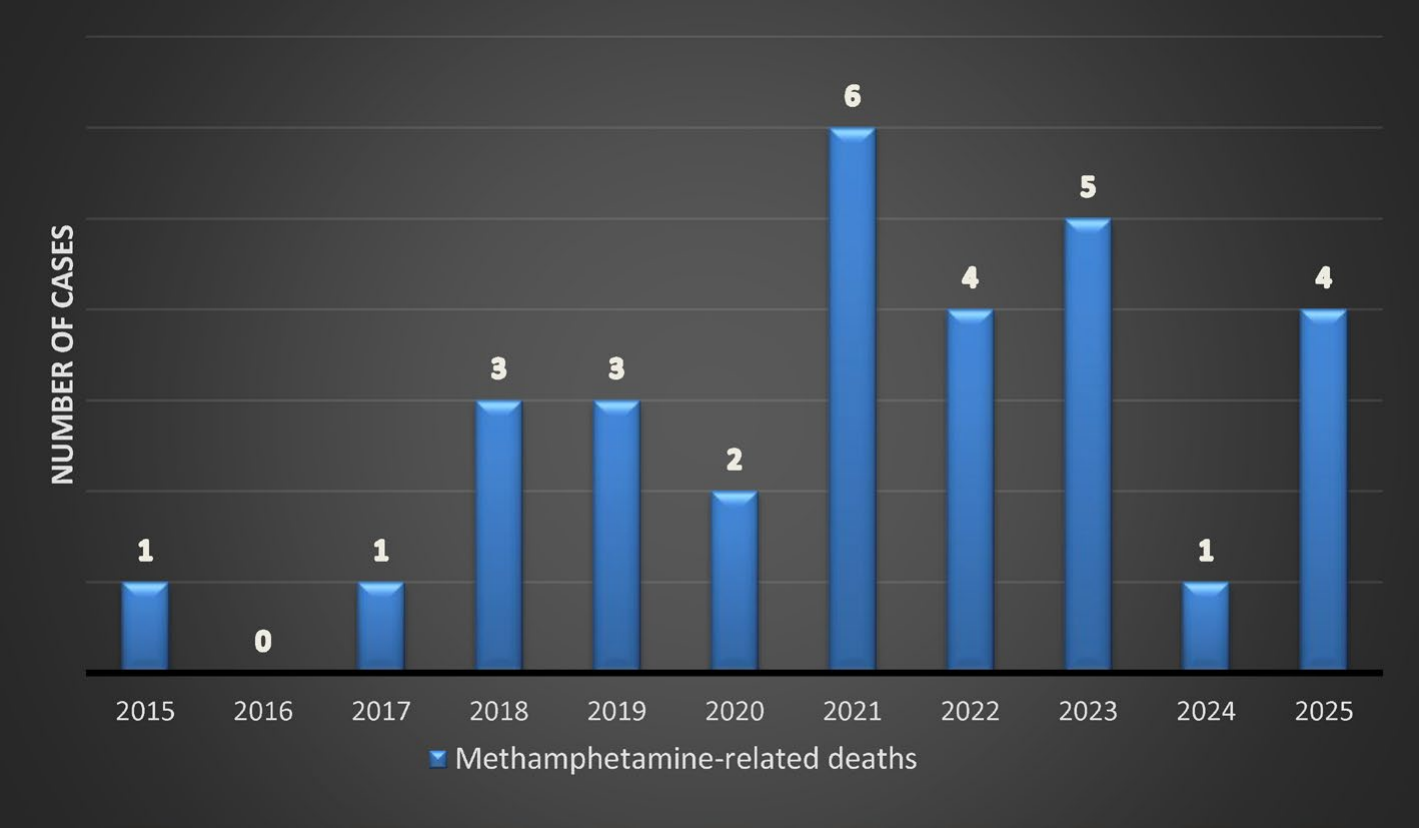
Annual number of methamphetamine‐related deaths (2015–2025).

From January 2015 to October 2025, 30 MA‐related deaths were registered in Zonguldak. The first case was recorded in mid‐2015 and the highest annual number of deaths was observed in 2021 (*n* = 6, 20.0%) (Figure 1). Overall, MA‐related deaths accounted for 1.61% of all forensic deaths during the study period. Annual case counts and proportions are presented in Table 1.

**TABLE 1:** Annual proportion (%) of methamphetamine‐related deaths among all forensic autopsies.

Year	All forensic deaths	Methamphetamine‐related deaths (% of all forensic autopsies)
2015	146	0.68
2016	86	0
2017	92	1.08
2018	208	1.44
2019	196	1.53
2020	198	1.01
2021	222	2.7
2022	200	2.0
2023	177	2.82
2024	184	0.54
2025	154	2.59
Total	1863	

The mean age was 33.4 ± 8.7 years; most cases were males (90.0%), and single or divorced (70.0%). Nearly half of the cases involved individuals living alone (*n* = 14). All deaths were single‐fatality events. The sociodemographic and clinical characteristics of the cases are summarised in Table 2.

**TABLE 2:** Sociodemographic and clinical characteristics of cases.

	$\bar{x}$ ± s
Age (years)	33.4 ± 8.7

	*n*	%
Sex		
Male	27	90.0
Female	3	10.0
Marital status		
Single	16	53.3
Married	9	30.0
Divorced	5	16.7
Living condition		
Alone	14	46.7
With family	16	53.3
Educational status		
Below primary school	5	16.7
Primary school	3	10.0
Above primary school	3	10.0
Unknown	19	63.3
Employment status		
Unemployed	10	33.3
Employed	10	33.3
Unknown	10	33.3
Incident location		
Home and annex	19	63.3
Public places	8	26.7
Workplace	2	6.7
In‐vehicle	1	3.3
Time of incident		
00:00–05:59	7	23.3
06:00–11:59	1	3.3
12:00–17:59	9	30.0
18:00–23:59	13	43.3
Primary cause of death		
Injuries^a^	14	46.7
Methamphetamine toxicity	9	30.0
Asphyxia by hanging	4	13.3
Cardiovascular disease	3	10.0
Medical intervention status		
No medical intervention	13	43.3
On‐site emergency medical services	5	16.7
In‐hospital	12	40.0
Location of death		
Scene of the incident	16	53.3
During the hospital period	14	46.7

^a^ Injuries included firearm injuries (*n* = 4), sharp force injuries (*n* = 3), traffic accidents (*n* = 3), electric shock (*n* = 2), falls from height (*n* = 1) and fire‐related injury (*n* = 1).

Among MA toxicity cases in which clinical findings were documented following medical intervention—either by on‐site emergency medical services or in hospitals—hyperthermia was observed in 5 cases and syncope was observed in 3 cases.

The mean years of potential life lost (YPLL) among the study cases was 41.6 ± 8.7 years. With respect to manner of death, accidental deaths were the most frequent (*n* = 17, 56.7%), followed by suicide (*n* = 7, 23.3%), natural death (*n* = 3, 10.0%) and homicide (*n* = 3, 10.0%). All natural deaths were attributed to cardiovascular causes, including acute coronary insufficiency (*n* = 2) and myocardial infarction (*n* = 1). Injury‐related causes of death occurred across accidental, suicidal and homicidal manners of death. Among the accidental deaths, MA toxicity was the leading cause (*n* = 9). Non‐natural deaths were more common than natural deaths.

Clinical and background characteristics included a history of self‐harm, drug dependence, alcohol use and psychiatric illness. Previous self‐inflicted scars were observed in 7 cases. A history of drug dependence was documented in 15 cases, while this information was unknown for the remaining cases. Alcohol use was reported in 7 cases. A history of psychiatric illness, including anxiety disorders (*n* = 6), major depressive disorder (*n* = 4) and schizophrenia (*n* = 2), was identified in 12 cases.

Peripheral blood MA concentrations ranged from 0.001 to 2.88 μg/mL, with a median concentration of 0.119 μg/mL. In 56.7% of the 30 MA‐related deaths, one or more additional substances were detected in postmortem blood samples, including psychoactive substances (e.g., opioids, THC and synthetic cannabinoids), ethanol and, in one case, elevated carboxyhemoglobin (Table 3).

**TABLE 3:** Postmortem examination findings.

*N* = 30			*n*	%
Pathological data	Body mass index (BMI)	Normal (BMI 18.5–24.9 kg/m^2^)	19	63.3
		Overweight (BMI 25.0–29.9 kg/m^2^)	8	26.7
		Obesity (BMI ≥ 30 kg/m^2^)	3	10.0
	Cardiac pathology^a^	Acute coronary insufficiency with coronary heart disease	2	6.7
		Myocardial infarction	1	3.3
Toxicological findings		Methamphetamine peripheral blood concentration (μg/mL) median (range)	0.119 (0.001–2.88)	
		*Other substances detected*	*n*	*%*
		Ethanol	8	26.7
		THC	4	13.3
		Synthetic cannabinoids	2	6.7
		Opiates	2	6.7
		Carboxyhemoglobin	1	3.3

^a^ Observed only in natural deaths attributed to cardiovascular causes.

## 4 Discussion

MA‐related deaths were observed throughout the study period, with annual case counts fluctuating over time. Given the limited sample size, these findings should be interpreted descriptively rather than as evidence of temporal trends. Nevertheless, the continued occurrence of MA‐related deaths over the study period underscores the ongoing forensic and public health relevance of MA use reported in previous studies [5, 14, 15].

The demographic profile of the cases in the present study (mean age 33.4 years; 90% male) is consistent with previous reports on MA‐related deaths, which consistently demonstrated a predominance of young adult males among fatal cases [4, 16, 17]. This finding is further supported by national data from Turkey, where 93.7% of drug‐related deaths in 2024 were male, with a mean age of 34.7 years [5]. Collectively, these findings indicate that MA‐related deaths disproportionately affect young males [18], likely reflecting higher stimulant use prevalence and increased vulnerability to high‐risk substance use patterns [19, 20, 21].

Chronic MA use is commonly associated with socioeconomically vulnerable profiles, including unstable housing and employment [14, 15, 17], which is consistent with the high proportion of individuals living alone and those who were single or divorced in our cohort. Previous studies have also reported a high prevalence of concomitant risk factors, including substance dependence, psychiatric comorbidity and violent behaviour, among MA‐related fatalities [14, 15, 17]. Collectively, these findings are consistent with the literature on MA‐related mortality.

The mean YPLL observed in this study reflects the substantial burden of premature mortality associated with MA use and aligns with global evidence indicating that drug use results in considerable losses of healthy life years [1]. In the Turkish context, where data on MA‐related mortality remain limited, these findings highlight a potentially underrecognised public health burden, particularly in regions undergoing industrial and socioeconomic transitions.

MA toxicity was the leading cause of accidental death in this cohort. These findings are consistent with the literature, which indicates that MA‐related deaths are predominantly unintentional in nature [15, 17]. Notably, despite ongoing prevention and regulatory efforts targeting illicit and controlled substances in Turkey, MA appears to be increasingly implicated in accidental drug‐related deaths, suggesting a shift in substance use patterns and an emerging public health concern. Natural deaths were relatively uncommon (10% of cases) and were exclusively attributed to cardiovascular causes, including acute coronary insufficiency and myocardial infarction, likely reflecting the well‐documented cardiotoxic effects of chronic stimulant use [22, 23, 24]. Nevertheless, MA‐related cardiovascular deaths may be underrepresented in forensic datasets, particularly in cases where stimulant involvement is not clinically suspected and toxicological analyses are not performed.

MA use is associated with euphoria, irritability, frontal disinhibition, reduced fatigue, tachycardia and hypertension. At higher doses, hyperthermia, seizures and life‐threatening cardiovascular complications may occur [22, 23]. In the present study, hyperthermia was observed in 5 cases and syncope was observed in 3 cases.

More than half of the MA‐related deaths in the present study involved concomitant detection of additional substances. Polydrug use is a well‐recognised feature of drug‐related fatalities and has been consistently reported in MA‐associated deaths [4, 25]. Previous studies have shown that concomitant use of MA with substances such as alcohol, opioids or other psychoactive agents may potentiate toxicity and increase the risk of fatal outcomes [4, 25, 26]. In this context, the high prevalence of multidrug exposure in our cohort is consistent with the broader literature on drug‐related mortality and further underscores the elevated mortality risk associated with combined substance use [27, 28].

Peripheral blood MA concentrations in the present series showed considerable variability. Interpretation of MA blood concentrations remains challenging in forensic practice, as toxic and lethal thresholds vary considerably across studies and are influenced by individual tolerance, chronic use patterns, polydrug exposure and postmortem redistribution [24, 29, 30, 31]. Previous reports have demonstrated substantial overlap between toxic, fatal and survivable MA concentrations, indicating that no universally applicable lethal threshold can be defined [29, 30, 32]. Even relatively low MA concentrations may trigger fatal cardiovascular or cerebrovascular complications–potentially through synergistic effects with other substances or underlying pathologies. Therefore, MA concentrations should be interpreted in conjunction with autopsy findings, toxicological context and case‐specific circumstances [33].

### 4.1 Limitations

Although forensic records and autopsy data enable more detailed analyses of the circumstances of death than general mortality registers do, this study has several limitations. Given the complexity of forensic investigations, some MA‐related deaths may not have been captured, as certain investigations may still be ongoing. This may lead to an underestimation of the actual number of MA‐related deaths. The relatively small sample size limited the interpretation of the findings. Therefore, the results should be interpreted as descriptive observations. One limitation of the present study was the lack of sociodemographic data, particularly concerning education and employment status. Nevertheless, incomplete background information may also reflect social vulnerability in forensic populations [34]. The study was conducted in Zonguldak, a province in the western Black Sea region of Turkey, which may limit the generalisability of the findings to other regions and international settings. Further large‐scale, multicenter forensic studies in Turkey are needed to characterise population‐level patterns of substance use and associated mortality more accurately.

### 4.2 Implications

The findings of this study highlight the substantial societal burden of MA‐related deaths and underscore the need for early intervention and rehabilitation strategies. To mitigate premature mortality associated with MA toxicity, targeted prevention strategies for high‐risk populations and comprehensive harm reduction approaches should be expanded. These may include improving access to evidence‐based substance use treatment, integrating mental health assessment and support into addiction services, and strengthening early identification and referral of individuals at increased risk of drug‐related harm. Strengthened collaboration among the healthcare, forensic and law enforcement sectors may improve efforts to address the growing burden of stimulant‐related harm.

## 5 Conclusion

In conclusion, this study provides regional forensic data on MA‐related deaths in Zonguldak, Turkey. Most decedents were young adult males and MA toxicity was the leading cause of accidental death. The mean YPLL demonstrates the considerable premature mortality associated with MA‐related deaths. These findings contribute to the limited forensic literature on MA‐related mortality in Turkey and provide a foundation for future multicenter forensic studies.

## Author Contributions

All authors approved the final version and certify that their contributions meet the standards of the International Committee of Medical Journal Editors.

## Funding

The authors have nothing to report.

## Conflicts of Interest

The authors declare no conflicts of interest.

## Data Availability

The data that support the findings of this study are available from the corresponding author upon reasonable request.
